# Aqueductal CSF stroke volume is associated with the burden of perivascular space enlargement in chronic adult hydrocephalus

**DOI:** 10.1038/s41598-024-63926-8

**Published:** 2024-06-05

**Authors:** Pasquale Gallina, Berardino Porfirio, Saverio Caini, Francesco Lolli, Antonio Scollato

**Affiliations:** 1 Neurosurgery Unit, CTO Hospital, Careggi University Hospital, Largo P Palagi 1, 50139 Florence, Italy; 2 Department of Neurosciences, Psychology, Drug Research and Child Health, University of Florence, Florence, Italy; 3 Department of Clinical and Experimental Biomedical Sciences “Mario Serio”, University of Florence, Florence, Italy; 4 Cancer Risk Factors and Lifestyle Epidemiology Unit, Institute for Cancer Research, Prevention, and Clinical Network, Florence, Italy; 5 Neurophysiology Unit, Careggi University Hospital, Florence, Italy; 6 Neurosurgery Unit, “Cardinale Panico” Hospital, Tricase, Lecce Italy

**Keywords:** Neurodegeneration, Hydrocephalus, Neurodegenerative diseases

## Abstract

The inflow of CSF into perivascular spaces (PVS) in the brain is crucial for clearing waste molecules. Inefficiency in PVS flow leads to neurodegeneration. Failure of PVS flushing is associated with CSF flow impairment in the intracranial hydrodynamic condition of CSF hypo-pulsatility. However, enlarged PVS (ePVS), a finding indicative of PVS flow dysfunction, is also present in patients with derangement of CSF dynamics characterized by CSF hyper-pulsatility, which increases CSF flow. Intriguingly, two opposite intracranial hydrodynamic conditions would lead to the same result of impairing the PVS flushing. To investigate this issue, we assessed the subsistence of a dysfunctional interplay between CSF and PVS flows and, if the case, the mechanisms preventing a hyper-pulsatile brain from providing an effective PVS flushing. We analyzed the association between phase contrast MRI aqueductal CSF stroke volume (aqSV), a proxy of CSF pulsatility, and the burden of ePVS in chronic adult hydrocephalus, a disease involving a broad spectrum of intracranial hydrodynamics disturbances. In the 147 (85 males, 62 females) patients, the age at diagnosis ranged between 28 and 88 years (median 73 years). Ninety-seven patients had tri-ventriculomegaly and 50 tetra-ventriculomegaly. According to the extent of ePVS, 113 patients had a high ePVS burden, while 34 had a low ePVS burden. aqSV, which ranged between 0 and 562 μL (median 86 μL), was increased with respect to healthy subjects. Patients presenting with less ePVS burden had higher aqSV (*p* < 0.002, corrected for the multiple comparisons) than those with higher ePVS burden. The present study confirmed the association between CSF dynamics and PVS flow disturbances and demonstrated this association in intracranial hyper-pulsatility. Further studies should investigate the association between PVS flow failure and CSF hypo- and hyper-pulsatility as responsible/co-responsible for glymphatic failure in other neurodegenerative diseases, particularly in diseases in which CSF disturbances can be corrected, as in chronic adult hydrocephalus.

## Introduction

The glymphatic system is a brain-wide fluid transport pathway that includes perivascular spaces (PVS) inflow of subarachnoid CSF followed by interstitial solute clearance^1^. Failure of PVS flow leads to the glymphatic system disruption, accumulation of interstitial waste molecules, and ultimately, neurodegeneration^2,3^.

Computational studies of intracranial dynamics have contributed to depicting the complex interplay of intracranial pressure, CSF pulsatile motion, glymphatic system fluid circulation, and brain movement (see Causemann et al.^4^ and references within).

The association between derangement of the CSF pulsatility (thought to help flush interstitial fluid through the glymphatic system^5^) in the context of intra-extracranial hydrodynamics disruption and PVS flow failure was previously hypothesized^6,7^. Indeed, Blair et al.^8^ demonstrated in cerebral small vessel diseases that decrease in foramen magnum CSF stroke volume, which is a proxy of CSF pulsatility^9^, resulted in less effective PVS flushing, as assessed by detection on MRI of enlarged PVS (ePVS), a finding related to stagnation of the fluid inside these spaces and indicative of PVS flow/function failure^8,10^. This demonstration was obtained in an intracranial hydrodynamic condition of CSF hypo-pulsatility^8^, which impairing the CSF flow, prevents/limits the same from reaching the sub-arachnoid spaces to be available for PVS pumping, the mechanism driven by arterial pulsatility allowing CSF to fill PVS and provide fluid motion^5^.

However, failure of PVS flow has also been observed in the opposite condition of CSF hyper-pulsatility as in adult hydrocephalus^10^, which showed ePVS^11^. This intriguing finding drove us to seek the subsistence of a dysfunctional interplay between CSF and PVS flows in the condition of increased CSF pulsatility and, if the case, to analyze mechanisms by which a CSF, vigorously pushed along its roots, ultimately fails in providing a flow adequate for PVS pumping.

To investigate this issue, we analyzed the burden of ePVS and its association with aqueductal CSF stroke volume (aqSV) by phase contrast MRI, which measures the mean volume of CSF passing through the aqueduct of Sylvius during a heart cycle^9^. aqSV assessment added to the comprehension of the pathophysiology of normal pressure hydrocephalus^12,13^. In the clinical settings, aqSV measurement has been proposed as a tool for CSF shunting selection of hydrocephalic patients^12^ (even if controversily^10^) and for post-shunting management^14,15^. aqSV is considered a proxy of CSF pulsatility^9,16,17^.

We assessed the association between aqSV and ePVS burden in patients with chronic adult hydrocephalus, which involves a large spectrum of ventriculomegaly^18^ expected to differ for age-related CSF pulsatility. In normal pressure hydrocephalus, a communicant tetra-ventriculomegaly of the elderly^19^, an increase of aqSV was observed in the initial phases of the disease due to the compression of the brain on enlarged ventricles leading to an increase of CSF pulsatility^13^. Afterward, aqSV decreased as a consequence of brain atrophy due to prolonged vascular damage^13^ or related to aging^20^. A more remarkable aqSV increase is expected in young patients because the compressive action on the dilated ventricles is operated by a vigorously pulsatile and trophic brain. Moreover, the presence among patients affected by chronic adult hydrocephalus also of cases with tri-ventriculomegaly, as well as of subjects suffering from secondary hydrocephalus, allowed us to assess our hypothesis in different intracranial hydrodynamic conditions related to increased resistance to CSF flow and asymmetric CSF accumulation between the supra- and infratentorial ventricular compartments.

## Results

**Figure 1:**
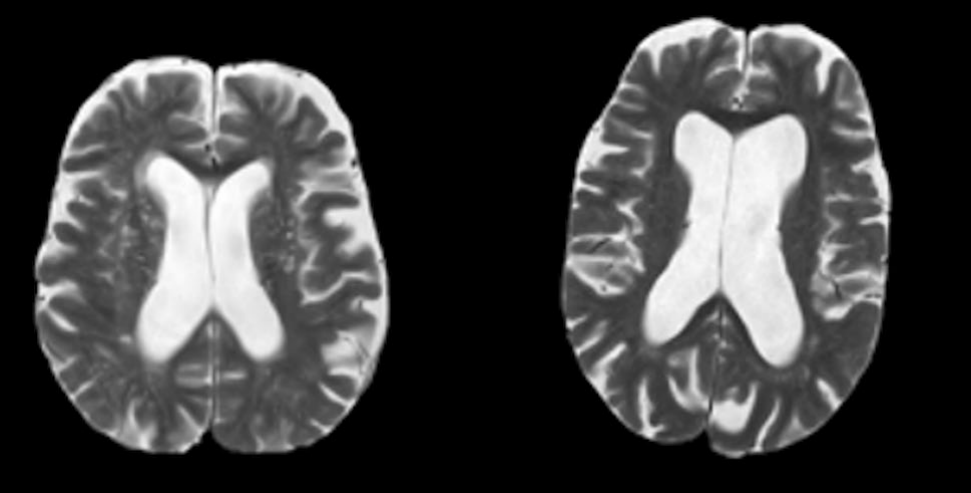
MRI visual assessment for enlarged perivascular spaces (ePVS) in two patients suffering from chronic adult hydrocephalus. ePVSs are seen on axial T2-weighted MRI, obtained at the level of the periventricular parenchyma, 20 mm above the anterior commissure, as linear-, ovoid- or round-shaped hyperintensities. The image on the left shows more than 11 ePVS (high ePVS burden), while the image on the right shows 10 or less ePVS (low ePVS burden).

One hundred forty-seven patients (85 males and 62 females) met the selection criteria. Age at diagnosis ranged between 28 and 88 years (median 73 years, IQR 14). Ninety-seven (66%) patients had tri-ventriculomegaly, while 50 (34%) had tetra-ventriculomegaly. One hundred and twenty-one (82.3%) patients suffered from hydrocephalus of unknown etiology and 26 (17.7%) from secondary hydrocephalus. The secondary etiology of hydrocephalus was related to brain tumor or brain tumor surgery in 9 cases, subarachnoid hemorrhage in 5 cases, severe brain trauma in 5 cases, cist of the pineal gland region in 3 cases, brain infection in 1 case, and brain ischemic stroke in 1 case. All the patients showed ePVS. According to the extent of ePVS, 113 patients (76.9%) had a high ePVS burden, while 34 (33.1%) had a low ePVS burden (Fig. 1). A velocity encoding of 15, 20, to 25 cm/s allowed aqSV measurement in all the patients without aliasing. aqSV ranged from 0 and 562 μL (median 86 μL, IQR 39 μL). There were no missing values for all the variables.

**Figure 2:**
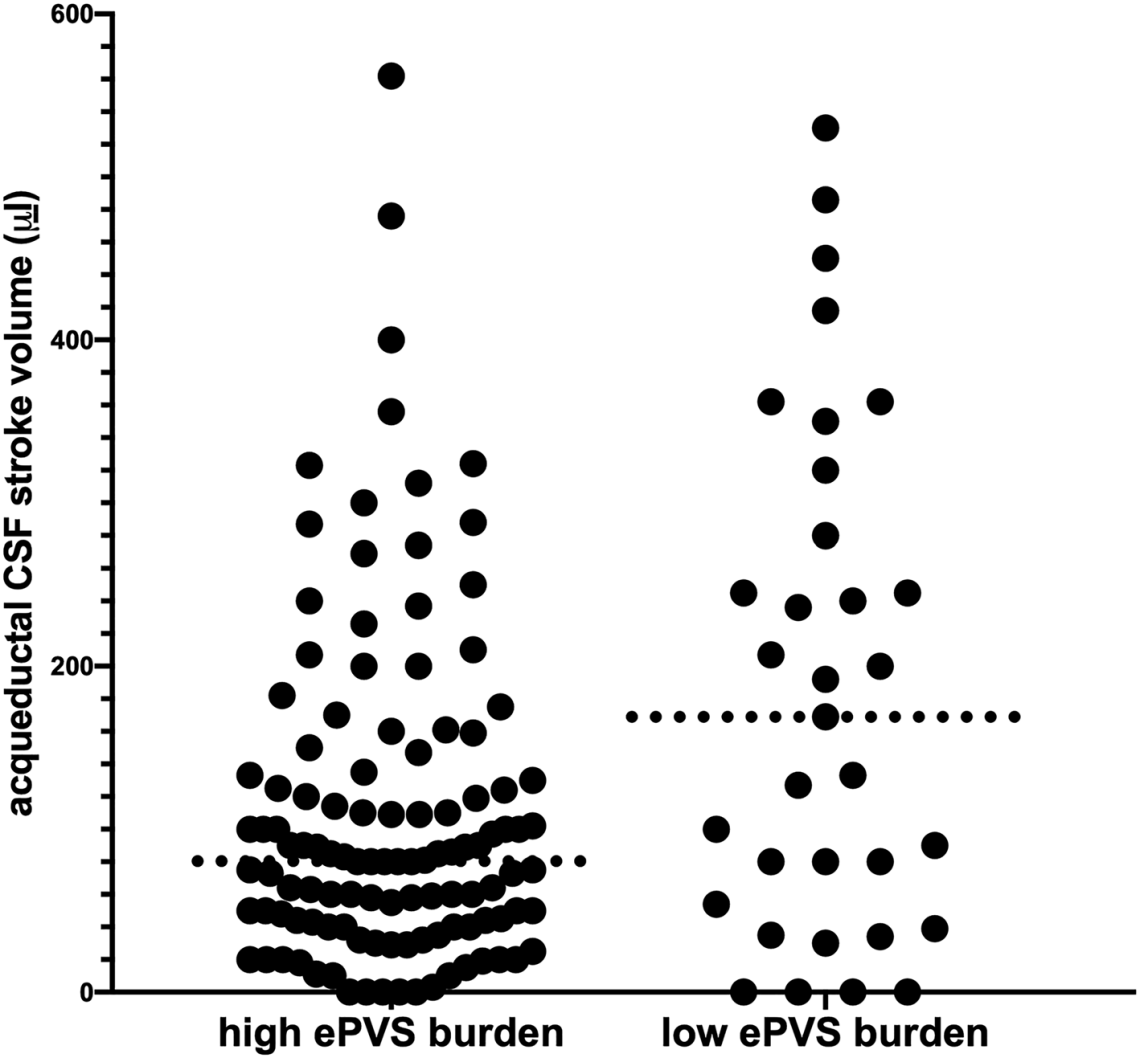
Individual values of aqueductal CSF stroke volume (aqSV) in 147 patients with chronic adult hydrocephalus with high (left) and low (right) burden of enlarged perivascular spaces (ePVS). The dotted lines represent median values. Patients with low ePVS burden (less than 11 ePVS at both basal ganglia and periventricular parenchyma) had significantly higher median aqSV (*p* = 0.02, adjusted for multiple comparisons) than patients with high ePVS burden (at least 11 ePVS or more at both basal ganglia and periventricular parenchyma).

The results were cross-tabulated for ePVS burden (Table 1), secondary versus unknown hydrocephalus (Table 2), and tri- versus tetra-ventricular hydrocephalus morphology (Table 3). A significantly higher median aqSV (*p* = 0.02, corrected for the multiple comparisons) was associated with patients presenting with a low burden of ePVS (Fig. 2). There were no differences in sex, age, hydrocephalus morphology, or etiology for ePVS burden (Table 1). We could not detect significant differences for any of the variables for hydrocephalus of unknown etiology versus secondary hydrocephalus (Table 2). Tetra-ventricular cases had higher median aqSV versus tri-ventricular cases (corrected *p* value, *p* < 0.01, Table 3). We analyzed the relation between median aqSV and sex, hydrocephalus morphology, hydrocephalus etiology, and ePVS burden in a multivariate linear regression (Table 4). Considering all the variables, a significant negative correlation of aqSV could be confirmed only with a low ePVS burden (*p* < 0.002, corrected for multiple comparison).

**Table 1:** Cross table for burden of enlarged perivascular spaces in 147 patients with chronic adult hydrocephalus.

Characteristic	N	Low ePVS burden, 34 (23%)	High ePVS burden, 113 (77%)	*p* value	q value^d^
Sex	147			0.034^b^	0.086
Females		9 (26%)	53 (47%)		
Males		25 (74%)	60 (53%)		
Hydrocephalus morphology	147			0.16^b^	0.26
Tetra-ventricular		15 (44%)	35 (31%)		
Tri-ventricular		19 (56%)	78 (69%)		
Aqueductal CSF stroke volume (μL)	147	158 (191)^a^	80 (93)	0.005^c^	0.024
Age at diagnosis (years)	147	72 (13)^a^	74 (12)	0.20^c^	0.26
Hydrocephalus etiology	147			0.31^b^	0.314
Secondary		8 (24%)	18 (16%)		
Unknown		26 (76%)	95 (84%)		

N: number. The patients were considered with a high enlarged perivascular spaces (ePVS) burden when at least 11 ePVS were found at both the levels of basal ganglia and of periventricular parenchyma or with low ePVS burden in the case of fewer ePVS at the same anatomic levels.

^a^Median (interquartile range).

^b^Pearson's Chi-squared test.

^c^Wilcoxon rank sum test.

^d^False discovery rate correction for multiple testing.

**Table 2:** Cross table for secondary versus. unknown etiology of ventriculomegaly in 147 patients with chronic adult hydrocephalus.

Characteristic	N	Secondary, 26 (18%)	Unknown, 121 (82%)	*p* value	q-value
Sex	147			0.99^b^	0.994^d^
Females		11 (42%)	51 (42%)		
Males		15 (58%)	70 (58%)		
Hydrocephalus morphology	147			0.19^b^	0.494^d^
Tetra-ventricular		6 (23%)	44 (36%)		
Tri-ventricular		20 (77%)	77 (64%)		
Aqueductal CSF stroke volume (μL)	147	78 (158)^a^	89 (127)	0.53^c^	0.66^d^
Age at diagnosis (years)	147	70 (16)^a^	74 (12)	0.20^c^	0.49^d^
Low burden of ePVS	147	8 (31%)	26 (21%)	0.31^b^	0.51^d^

N: number. The patients were considered with a low enlarged perivascular spaces (ePVS) burden when less than 11 ePVS were found at both the levels of basal ganglia and of periventricular parenchyma.

^a^Median (interquartile range).

^b^Pearson's Chi-squared test.

^c^Wilcoxon rank sum test.

^d^False discovery rate correction for multiple testing.

**Table 3:** Cross table for tri- versus tetra-ventricular morphology of ventriculomegaly in 147 patients with chronic adult hydrocephalus.

Characteristic	N	Overall, 147	Tetra, 50 (34%)	Tri, 97 (66%)	*p* value	q-value^d^
sex	147				0.46^b^	0.46
Females		62 /147 (42%)	19/50 (38%)	43/97 (44%)		
Males		85/147 (58%)	31/50 (62%)	54/97 (56%)		
Aqueductal stroke volume (μL)	147	127 (120)^a^	156 (117)	113(119)	0.02^c^	0.010
Age at diagnosis (years)	147	70.5 (12)^a^	70.8 (14)	70.3(11.2)	0.24^c^	0.30
Hydrocephalus etiology	147				0.19^b^	0.30
Secondary		26/147 (18%)	6/50 (12%)	20/97 (21%)		
Unknown		121/147 (82%)	44/50 (88%)	77/97 (79%)		
Low burden of ePVS	147	113/147 (77%)	15/ 50 (30%)	19/97 (20%)	0.16^b^	0.30

N: number of patients. The patients were considered with a low enlarged perivascular spaces (ePVS) burden when less than 11 ePVS were found at both the levels of basal ganglia and of periventricular parenchyma.

^a^Median (interquartile range).

^b^Pearson's Chi-squared test.

^c^Wilcoxon rank sum test.

^d^False discovery rate correction for multiple testing.

**Table 4:** Multivariate linear regression of aqueductal CSF stroke volume for sex, hydrocephalus morphology, age at diagnosis, hydrocephalus etiology, and burden of enlarged perivascular spaces in 147 patients with chronic adult hydrocephalus.

Predictors	Coefficient	SE	t-value	*p* value
Intercept	191.3379	62.786	3.0475	0.003
Sex				
Males–females	18.9741	19.692	0.9635	0.337
Hydrocephalus morphology				
Tri-ventricular–tetra-ventricular	− 33.6577	20.493	− 1.6424	0.103
Age at diagnosis (years)	− 0.0341	0.796	− 0.0429	0.966
Hydrocephalus etiology				
Unknown–secondary	7.1854	25.499	0.2818	0.779
Burden of ePVS				
High–low	− 72.8090	23.382	− 3.1140	0.002

The patients were considered with high enlarged perivascular spaces (ePVS) burden when at least 11 ePVS were found at both the levels of basal ganglia and of periventricular parenchyma, or with a low ePVS burden in the case of fewer ePVS at the same anatomic levels.

## Discussion

PVS flow failure was a common finding in our chronic adult hydrocephalic patients, in whom the CSF derangement underlining the disease led to CSF hyper-pulsatility. The extent of the latter was associated with the burden of the PVS flow failure.

All the patients had the disruption of the PVS flow. Abnormality of CSF dynamics as responsible for the failure of the PVS motion has been reported in terms of CSF hypo-pulsatility^8^. In our model, PVS flow failure occurred in the opposite condition of CSF hyper-pulsatility, as indicated by the increased aqSV of the whole population (median 86 μL), as compared to the value of aqSV normality, which ranges between 30 and 50 μL^21–23^. In patients with normal pressure hydrocephalus, higher aqSV (103.5 μL) as compared to healthy subjects has been previously observed by Shanks et al.^24^. In normal pressure hydrocephalus, increased aqSV of a similar extent respect to that of the above-mentioned paper^24^ was also previously reported by our group^13–15^.

The present study, which expanded aqSV assessment to tri-ventricular hydrocephalus, supported that ventricular dilatation developing in the closed skull increases the ability of the brain to squeeze the supra-tentorial ventricles, thus contributing to vigorously moving the CSF along its roots^13^. In this intracranial hydrodynamics of increased CSF pulsatility, the double CSF pulsatility extent was necessary to partially preserve PVS flow with respect to the cases with a considerable PVS flow disruption (median aqSV was 158 μL in the patients with low ePVS burden, and 80 μL in the patients with high ePVS burden, *p* = 0.005).

The extent of aqSV was the only variable associated with less or more preservation of the PVS flow. In particular, contrary to our thoughts, the extent of aqSV was not age-related. However, the smaller number of tetra-ventricular cases than the tri-ventricular ones may have represented a selection bias influencing the result. The larger recruitment of patients with tri-ventriculomegaly was due to the Author’s clinical practice of a nonsystematic aqSV measurement in older tetra-ventricular cases with high ePVS burden, who were considered less suitable for CSF shunting—the gold standard in their treatment^25^—when they had already developed atrophy^26^ and imaging signs of ongoing neurodegeneration^27^.

The patients with tetra-ventriculomegaly showed higher median aqSV than those with tri-ventricular involvement (median aqSV 156 vs. 113 μL, respectively, *p* = 0.02). In the absence of a difference in age between the patients with tetra-ventriculomegaly and those with tri-ventriculomegaly (75 vs. 72 years, respectively, *p* = 0.23), the finding could make you think a less CSF pulsatility of the latter’s brain. In fact, in the patients with tri-ventricular hydrocephalus, median aqSV was lowered by the presence of cases with aqSV equal to 0 μL or very low. This happened in the presence of overwhelming/high anatomical/functional aqueductal resistances (data not shown) and is not indicative of hypo-of pulsatility of some tri-ventricular hydrocephalus. This consideration brings out the role of aqueductal resistances, which impairing CSF flow deranged the PVS fluid motion. Note that, some patients with aqSV among the highest of the series (up to 562 μL, a value which is about ten times more respect to the aqSV of healthy subjects^21–24^, and about five times more respect to the aqSV of tetra-ventricular hydrocephalus^13–15,24^) have not been spared from high ePVS burden*.* Therefore, we can hypothesize that in neurodegenerative disease, the extent of CSF pulsatility to provide maximum preservation of the PVS flow is not absolute, but relative to specific derangements of the CSF flow which hit the glympathic system.

The issue of hydraulic resistances along CSF roots as a factor influencing the PVS flow in chronic adult hydrocephalus would merit further analysis, even if our study failed to demonstrate the relevance of this factor when considering the hydrocephalus etiology. The high ePVS burden was not different between secondary hydrocephalus and hydrocephalus of unknown etiology. Instead, we expected that in the firsts, alteration of CSF roots, making the CSF flow more challenging, would correspond to more significant disruption of the PVS flow. However, the criterion followed in considering hydrocephalus as secondary was very stringent (see population and methods), possibly resulting in a numeric overestimation of the hydrocephalus of unknown etiology, thus undermining the statistical analysis.

In the patients who showed high ePVS burden, the current aqSV was matched to an already established pathological situation. Without information about the time of PVS enlargement, the aqSV might not reflect the intracranial hydrodynamic condition presented by the patient when PVS motion failed. This consideration made it impossible to find thresholds of aqSV anticipating PVS enlargement. Moreover, the conclusions drawn from the patients with high ePVS burden must be taken cautiously, as other factors not measured in this study might be responsible/co-responsible for derangement in PVS flow.

We cannot infer whether effective CSF pulsatility makes subarachnoid CSF available for PVS pumping^5^ or if it also contributes to driving PVS filling/flushing. However, our study has indicated a much more relevant role than currently thought of CSF flow disturbances in PVS flow and should promote a reflection on the hierarchy of intracranial drivers of fluid movement. Cerebral arterial pulsation, respiratory motion, constant production of CSF, and sleep, which are considered responsible for PVS flow, are also the drivers of CSF flow^11^. Thus, we wonder if the supposed PVS flow drivers operate as such or if they are promotors of actions converging in a unique pathway that determines an effective CSF pulsatility. Novel imaging methods providing direct evaluation of CSF and PVS flows, and their relationship with arterial pulsation^28^, promise to clarify the issue.

In addition to the above-mentioned possible bias in the selection of the patients, a limitation of our study lies in its retrospective design. Longitudinally, assessments of the correlation between the number, size, and site of ePVS^29^ with aqSV would add to the comprehension of the anatomical and functional connection between macroscopic CSF routes and glymphatic motion in chronic adult hydrocephalus. Secondary hydrocephalus represents a model to investigate the chronological interplay between the development of ventriculomegaly and PVS enlargement by following patients who experience brain injuries possibly related to the development of ventriculomegaly^18^. In the clinical setting, it might be helpful to assess the association between aqSV and high ePVS burden as a predictor of CSF shunting failure or of unsatisfying and temporary benefit in chronic adult hydrocephalus, particularly on dementia, for which glymphatic dysfunction is instrumental^30^.

The association between aqSV and ePVS in non-hydrocephalic patients would strengthen the idea of a common substrate of CSF flow disturbances in neurodegenerative disorders^6^ in different conditions of CSF pulsatility. It would be crucial to establish thresholds of aqSV for specific pathological conditions above which the risk of PVS flow dysfunction becomes actual, to possibly prevent the glymphatic system failure.

### Patients and methods

All methods were performed following the relevant guidelines and regulations. This observational study was approved by the Area Vasta Centro Ethics Committee (approval number 22023). We reviewed (December 2023) the already existing data, without any further intervention, of a population of patients with chronic adult hydrocephalus who were admitted (between February 2013 and December 2023) to the Neurosurgery Units of Careggi University Hospital, Florence, Italy, and “Cardinale Panico” Hospital, Tricase (Lecce), Italy. We obtained informed consent for clinical procedures from all patients. Data were anonymized before analysis. Management of hydrocephalic patients has previously been reported^31^.

Patients with MRI assessment and aqSV evaluation were included in the study. Patients with congenital, pediatric, and adolescent onset of hydrocephalus were excluded. The patients with acute hydrocephalus, i.e., secondary hydrocephalus developing within 4 months from the brain injury responsible for the ventricular dilatation, were excluded.

aqSV, i.e., the mean volume of CSF passing through the Silvius aqueduct during a heart cycle, was calculated by phase contrast MRI (for an overview of aqSV, see Yamada et al.^9^). Images were obtained with 1.5 T superconductive MRI scanners using phase-contrast cine MRI pulse sequence (TR, 24 ms; TE, 15 ms; flip angle, 15°; number of excitations, 2; matrix, 256 × 256 pixels, section thickness, 4 mm). A cine acquisition with sensitivity to velocity in the section-select direction was obtained on an oblique axial plane of section perpendicular to the aqueduct. Foe each patient, we tried to use the same velocity encoding value or adjusted it to null the aliasing effect. Retrospective cardiac synchronization was used to gather flow information within the complete cardiac cycle (peripheral pulse unit triggering). For further details on measurement of aqSV, see Scollato et al.^13^.

ePVSs were identified on T2-weighted MRI as linear-, ovoid- or round-shaped (depending on the slice direction) hyperintensities. They were considered enlarged when their diameter was ≥ 1 mm. We analyzed ePVS at the basal ganglia and periventricular parenchyma levels on a predefined slice. For basal ganglia, this was the slice showing the anterior commissure or, when not visible, the first slice above it. The slice for assessment of ePVS at the level of the periventricular parenchyma was obtained 20 mm (± 4) above the anterior commissure. The extent of ePVSs was rated in four classes according to the visual rating scale proposed by Wardlaw et al.^11^. The patients were considered to have a high ePVS burden when at least 11 enlarged PVSs were found on both anatomical sites, or they had a low ePVS burden in the case of fewer.

Tri- or tetra-ventriculomegaly was assessed on T2-weighted MRI on a verticofrontal section at the level of the anterior commissure and a sagittal section at the level of the inter-commissural line.

Hydrocephalus was strictly considered secondary in the presence of previous imaging demonstration of the absence of ventriculomegaly and the occurrence of ventricular dilatation after the brain insult. In the absence of this demonstration, hydrocephalus was labeled as having an unknown etiology.

### Statistic methods

The Shapiro–Wilk test assessed normality. We employed non-parametric tests for variables with non-normal distribution. Differences in distribution were considered significant with a p-value < 0.05 using the Mann–Whitney U test for continuous variables and the chi-square test for categorical variables. Q-values considered the false discovery rate. A multiple linear regression assessed the linear relationship between continuous (age) and categorical explanatory variables (sex, hydrocephalus morphology, hydrocephalus etiology, and ePVS burden) and the single continuous response variable aqSV. We conducted all analyses with Stata Software Release 14, Wizard for MAC (Evan Miller®), and Jamovi.

## Data Availability

The datasets generated and/or analyzed during the current study are not publicly available due to participants’ privacy protection but are available from the corresponding authors upon reasonable request.
